# Effect of OH scavengers on the chemical composition of α-pinene secondary organic aerosol†

**DOI:** 10.1039/d2ea00105e

**Published:** 2022-11-04

**Authors:** David M. Bell, Veronika Pospisilova, Felipe Lopez-Hilfiker, Amelie Bertrand, Mao Xiao, Xueqin Zhou, Wei Huang, Dongyu S. Wang, Chuan Ping Lee, Josef Dommen, Urs Baltensperger, Andre S. H. Prevot, Imad El Haddad, Jay G. Slowik

**Affiliations:** a Laboratory of Atmospheric Chemistry, Paul Scherrer Institute 5232 Villigen Switzerland david.bell@psi.ch jay.slowik@psi.ch; b Tofwerk 3600 Thun Switzerland; c Institute of Meteorology and Climate Research, Karlsruhe Institute of Technology 76344 Eggenstein-Leopoldshafen Germany

## Abstract

OH scavengers are extensively used in studies of secondary organic aerosol (SOA) because they create an idealized environment where only a single oxidation pathway is occurring. Here, we present a detailed molecular characterization of SOA produced from α-pinene + O_3_ with a variety of OH scavengers using the extractive electrospray time-of-flight mass spectrometer in our atmospheric simulation chamber, which is complemented by characterizing the gas phase composition in flow reactor experiments. Under our experimental conditions, radical chemistry largely controls the composition of SOA. Besides playing their desired role in suppressing the reaction of α-pinene with OH, OH scavengers alter the reaction pathways of radicals produced from α-pinene + O_3_. This involves changing the HO_2_ : RO_2_ ratio, the identity of the RO_2_ radicals present, and the RO_2_ major sinks. As a result, the use of the OH scavengers has significant effects on the composition of SOA, including inclusions of scavenger molecules in SOA, the promotion of fragmentation reactions, and depletion of dimers formed *via* α-pinene RO_2_–RO_2_ reactions. To date fragmentation reactions and inclusion of OH scavenger products into secondary organic aerosol have not been reported in atmospheric simulation chamber studies. Therefore, care should be considered if and when to use an OH scavenger during experiments.

## Abstract 2

We present a detailed molecular characterization of SOA produced from α-pinene + O_3_ with a variety of OH scavengers. Radical chemistry controls the composition of SOA and scavengers alter these pathways and are incorporated into the SOA.

## Graphical abstract

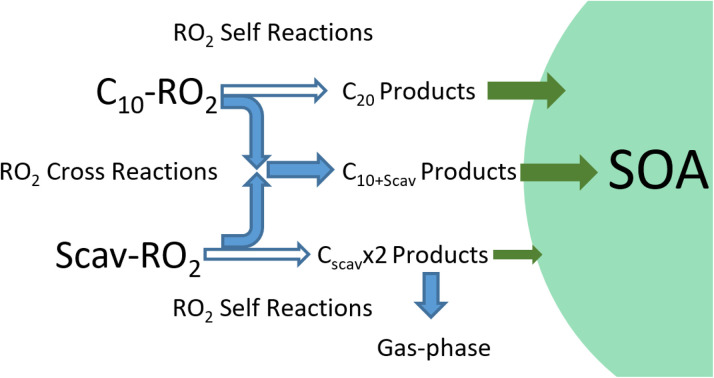

**Environmental significance**

Secondary organic aerosol (SOA) is a major component of atmospheric particles, and is often simulated using laboratory studies in smog chambers. OH scavengers are a common additive to smog chambers, when reactions by other oxidants are investigated. OH scavengers are small organic molecules that possess too high volatility to contribute by themselves to formation of SOA. In this work, we demonstrate the impact of OH scavengers on the radical balance in smog chambers and its inclusion into SOA, which substantially alters SOA composition. This can have strong impacts on SOA yield parametrizations, volatility distribution determination and potentially the assessments of SOA toxicity and climate impacts. Therefore, the use of OH scavengers does not necessarily faithfully reproduce the processes occurring in the atmosphere.

## 1 Introduction

Organic aerosol (OA) makes up between 20–90% of the global aerosol burden.^1^ Much of the OA in the atmosphere results from oxidation processes forming secondary species (secondary organic aerosol, SOA) that have low volatility, so after the transformation the formed molecules are more likely found in the particle phase than the gas phase.^2^ Quantitative investigation of these processes in the atmosphere is often impractical due to the large number of precursor volatile organic compounds (VOCs) and possible reaction pathways. Therefore, comparison of ambient measurements to laboratory studies provides the opportunity to study single VOCs oxidized under controlled conditions, thus disentangling complexities present in the atmosphere.^3^ Monoterpenes are prevalent VOC precursors and yield a substantial fraction of SOA globally, making them a frequent target for laboratory studies.^4^

Perhaps the most studied ideal system in the laboratory is the ozonolysis of α-pinene. Ozonolysis of alkenes also produces OH radicals with yields up to 115%,^5^ providing a competing oxidation pathway which obscures the desired investigation of pure ozonolysis.^6^ For example, during α-pinene ozonolysis experiments, up to half of the α-pinene is estimated to react with OH.^7^ Therefore, the use of OH radical scavengers is standard practice to limit the oxidation to a single pathway and determine the corresponding yields of SOA formation,^7–12^ physical properties,^13,14^ and composition.^9,11,15–17^ The yields of SOA formation have been extensively studied with scavengers and have been found to vary substantially based on the scavenger identity, where small molecules (*e.g.* methanol, formaldehyde, propanol) have lower yields of SOA formation than cyclohexane.^11^ Overall, the use of a scavenger implicitly assumes that the scavenger exerts a negligible effect on SOA composition and properties.

By preventing the reaction of α-pinene with OH *via* an OH scavenger, a consequence is to limit the formation of OH oxidation products. While the reaction of α-pinene with O_3_ mainly produces C_10_H_15_O_4,6_ peroxy-radicals (RO_2_), in reactions with OH radicals the main radicals formed are C_10_H_17_O_3,5_.^18,19^ Therefore, studies have reported the reduction of highly oxygenated molecules (HOMs) produced through the C_10_H_17_O_*x*_ radical pathway, in the presence of an OH scavenger.^18,20^ However, as an undesired effect, the use of an OH scavenger also alters the fate of the RO_2_ radicals produced from ozonolysis and consequently the chemical composition of the resulting SOA. In the absence of nitrogen oxide (NO), RO_2_ radicals react with HO_2_ to form an alkoxy radical (RO) or hydroperoxides, or with other RO_2_ radicals to form an RO radical or different closed shell molecules, including ROOR′ dimers (see ESI†).^19,21,22^ The pathway to ROOR′ dimers is particularly important because these products have low volatility and are expected to be a major fraction of SOA.^19,23–25^ Changes in RO_2_ chemistry due to the use of scavengers alters the formation rates of dimers and the ability of RO_2_ radicals to undergo autoxidation, and these effects on SOA composition require systematic evaluation. On the one hand, the presence of some OH scavengers (*e.g.* H_2_, CO, alcohols, *etc.*) alters the HO_2_ : RO_2_ ratio,^10,11^ which may have an effect on dimer yields. On the other hand, the identity of the RO_2_ radicals formed will change because of the presence of a scavenger (*e.g.* cyclohexane, *etc.*), by replacing the RO_2_ radicals formed from the α-pinene + OH pathway with the scavenger + OH oxidation pathway. The products of the reaction between two RO_2_ radicals are strongly dependent on the radical structure, where the reaction branching ratios are in favor of dimer formation for larger radicals.^18,19,21,26,27^ Therefore, while RO_2_ radicals formed from small scavengers are expected to decrease dimer formation, radicals from larger scavengers are expected to recombine with the radicals from the VOC of interest forming mixed dimers. The latter have been observed in flow tube reactors^18,26^ and in the gas phase in smog chamber studies,^27^ but particle phase observations are currently lacking. Accordingly, OH scavengers can affect many aspects of the oxidation process, and their ubiquitous use in the atmospheric community necessitates the study of their effects on SOA formation and its composition.

Here, we explore the chemical changes in α-pinene SOA forming in the presence and absence of different OH scavengers (butanol, cyclopentane, and cyclohexane). We utilize the extractive electrospray ionization time-of-flight mass spectrometer (EESI-TOF),^28^ as a soft ionization technique, to probe the changes on a molecular level. To understand the gas phase reactions leading to the observed molecules in the particle phase, we complement the SOA studies by also using a flow reactor.

## 2 Experimental

Studies were performed in Teflon atmospheric simulation chambers (27 m^3^ or 8 m^3^) at the Paul Scherrer Institute.^29,30^ The chambers are housed in temperature-controlled enclosures maintained at 20 ± 1 °C. The relative humidity (RH) for each experiment was 50%. Instrumentation included a proton-transfer mass spectrometer (PTR-MS, PTR-TOF-8000, Ionicon), an EESI-TOF including an atmospheric pressure time-of-flight mass spectrometer (Tofwerk), a scanning mobility particle sizer (SMPS, TSI model 3938) and an ozone gas monitor (Thermo 49C). Experiments were performed by injecting ozone into the chamber (200–500 ppb), followed by injection of an OH scavenger (if used), and then α-pinene (see Table S1† for details). Gas phase concentrations were monitored by a PTR-MS and for selected experiments a NO_3_-chemical ionization mass spectrometer (NO_3_-CIMS). The OH scavengers utilized were *n*-butanol (∼100 ppm), cyclopentane (200 ppm), and cyclohexane (200 ppm). These concentrations of OH scavenger resulted in OH reacting with the scavenger 99.9% of the time for all scavengers. The EESI-TOF provides highly time-resolved measurements (1 Hz) of the SOA molecular ions. The aerosol flow is continuously sampled and intersects with a spray of charged droplets doped with ∼100 ppm of NaI generated by a conventional fused silica electrospray capillary. The water-soluble portion of the aerosol is extracted into the droplets, which then yields intact SOA molecules in the form of Na^+^-adducts. Prior to interaction with the electrospray, a multi-walled charcoal denuder strips the gas phase constituents and leaves the aerosol, alone, to interact with the electrospray. The aerosol sample was regularly switched to a filter blank (4 min sample and 1 min filter) throughout the experiment to obtain regular background measurements. Detailed descriptions of the instrument can be found in lab studies,^28,31^ as well as in field studies.^32,33^ The particle phase mass concentrations were calculated using the size distributions obtained by the SMPS using a density of 1.2 g cm^−3^.^34^ The maximum mass concentrations are reported in Table 1 and are between 23–28 μg m^−3^ when no scavenger is present. The mass loadings are 12–16 μg m^−3^ when OH scavengers are used, similar to the reductions observed in Iinuma *et al.*^9^

**Table 1:** Experimental conditions explored with the flow tube and atmospheric simulation chamber

Experiment #	Scavenger	Experimental setup	α-Pinene (ppb)	Scavenger (ppm)	O_3_ (ppb)	Mass loading (μg m^−3^)
1	No	Smog chamber	25	—	160	23
2	No	Smog chamber	25	—	225	28
3	No	Flow tube	10	—	5000	—
4	Butanol	Smog chamber	25	200	250	16
5	Butanol	Flow tube	10	200	5000	—
6	Cyclohexane	Smog chamber	25	200	230	15
7	Cyclohexane	Flow tube	10	200	5000	—
8	Cyclopentane	Smog chamber	25	200	200	12

Flow-tube experiments were also performed in a ∼5 L glass vessel with a total flow rate of 20 L min^−1^, resulting in a residence time of ∼12 seconds at an RH of ∼5%. A constant source of α-pinene and ozone was injected into the flow tube, periodically an OH scavenger was additionally injected into the flow tube while maintaining a constant flow rate (see Table 1 for conditions). A condensation particle counter (CPC, TSI 3776, lower cut off 2.5 nm) continually monitored the particle number concentration and showed no particle formation in any experiment. During the flow-tube experiments the multi-channel denuder was removed from the EESI-TOF and the direct gas-phase products were detected. Backgrounds were assessed by comparing the signal observed by the EESI-TOF when only zero air was being passed through the flow tube both before and after each experiment, to achieve background levels. As mentioned above the total flow rate of the flow tube was 20 L min^−1^ and the flow rate from the flow tube to the EESI-TOF (∼0.5 m) was 10 L min^−1^, while the EESI-TOF sampled at 1 L min^−1^*via* a 3 cm long stainless steel tube. Below, the flow tube data is used to explore relative changes in the composition, and to verify products that are formed in the gas phase resulting from interactions between OH scavengers and α-pinene oxidation products. Therefore, absolute concentrations and reaction rates were not obtained. The experiments here were modelled with a 0-D box model (F0AM)^35^ using the chemical mechanism in MCM 3.3.1.^36,37^

### 3.1 Experimental results

**Fig. 1:**
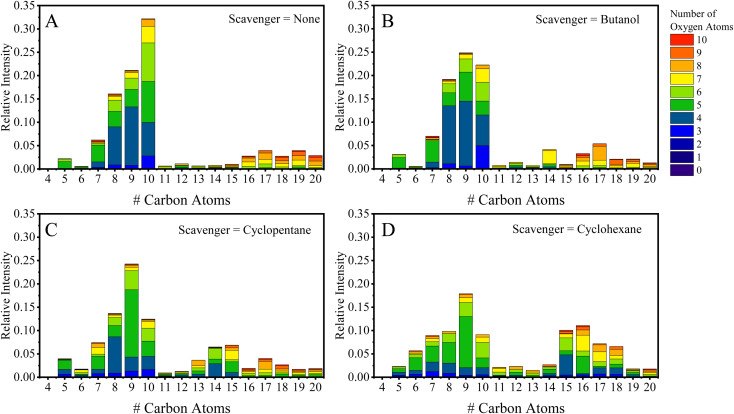
(A–D) Data from EESI-TOF binned according to number of carbon atoms (*x*-axis) and number of oxygen atoms at the time of maximum SOA mass concentration during an α-pinene ozonolysis experiment with (A) no scavenger present, (B) butanol present, (C) cyclopentane present, and (D) cyclohexane present.

Previous work performed in our chambers utilizing α-pinene SOA, in Pospisilova *et al.*, showed the composition of α-pinene to be highly time-dependent, and products from OH chemistry (C_10_H_18_O_*x*_ molecules) were found to be especially reactive with lifetimes below 30 minutes.^31^ Due to the complex evolution in composition, we will initially discuss EESI-TOF composition measurements performed at two experimental times: (1) within the first 30 minutes of the experiment; and (2) at the maximum of SOA mass. Fig. 1A–D show the carbon distribution at maximum mass with the bars colored by the number of oxygen atoms present for experiments without an OH scavenger, and with butanol, cyclopentane, and cyclohexane as OH scavengers. Fig. 1A shows the typical composition of α-pinene SOA formed without a scavenger at the time of maximum mass, binned in terms of the number of C and O atoms (*x*-axis and colors, respectively). Overall, C_5_–C_10_ molecules dominate in the monomer region and C_14_–C_20_ molecules in the dimer region, which together represent more than 90% of the total EESI-TOF signal observed in all scavenger-free experiments. The largest fraction of molecules formed contains 10 carbon atoms, and the hydrogen distribution for the C_10_ species consists mainly of H = 14, 16, and 18. Previous work shows it is possible to separate the contribution of different oxidation schemes (OH *vs.* O_3_) (*e.g.*, C_10_H_14_O_*x*_ and C_10_H_16_O_*x*_ are formed from O_3_ chemistry, while C_10_H_16_O_*x*_ and C_10_H_18_O_*x*_ come from OH chemistry).^18,19^ Therefore, the C_10_H_18_O_*x*_ molecules can be used as an initial assessment for whether or not the OH chemistry pathway is depleted.

**Fig. 2:**
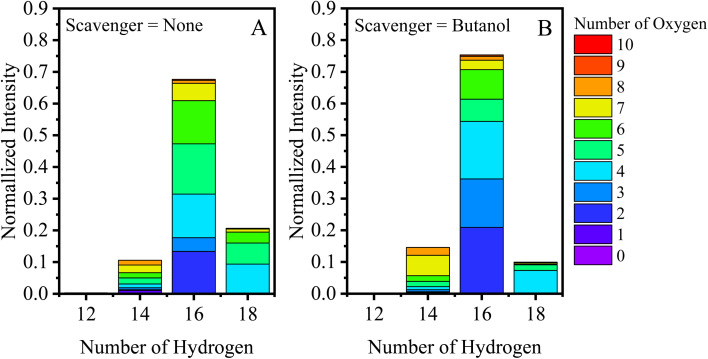
Hydrogen distribution plotted for C_10_H_*x*_O_*y*_ molecules (30 min after α-pinene addition), and colored according to the number of oxygen atoms present for an experiment (A) without an OH scavenger, and (B) with butanol present as a scavenger.

Fig. 2A and B break down the C_10_ species observed by the EESI-TOF in terms of number of hydrogen atoms (H = 12, 14, 16, and 18) with number of oxygen atoms between 2–10 in experiments without an OH scavenger and butanol as an OH scavenger, respectively, 30 min after the addition of α-pinene. The C_10_H_18_O_*x*_ fraction without the scavenger is ∼25% of the total C_10_ contribution, consisting of O_4_–O_7_ molecules (Fig. 2A), while the butanol scavenger C_10_H_18_O_*x*_ fraction is only ∼15% (Fig. 2B). Overall, the fraction of C_10_H_18_O_*x*_ is significantly reduced for the latter case, and instead of spanning #O = 4–7, C_10_H_18_O_4_ is almost exclusively formed. C_10_H_18_O_4_ was found previously to decay away quickly in the particle phase, likely due to its high reactivity.^31^ While the majority of C_10_H_18_O_*x*_ molecules are formed through OH chemistry, C_10_H_18_O_4_ can also be formed *via* the reaction of the Criegee intermediate with H_2_O.^15,19^ Additionally, the change associated with the scavenger demonstrates that the C_10_H_18_O_*x*_ molecules (*x* = 5–7) are not a result of water clusters with C_10_H_16_O_*x*_ molecules, but rather formed *via* OH chemistry. Results when using cyclohexane as a scavenger are included in the ESI (Fig. S1A†), and agree with the results shown in Fig. 2, while formation of dimers *via* cyclopentane oxidation products complicates the analysis for that system (Fig. S1B†).

Fig. 1B–D show the carbon distribution at maximum mass when butanol (1B), cyclopentane (1C), and cyclohexane (1D) were used as OH scavengers. Comparing Fig. 1A (no scavenger) to 1B (butanol scavenger), there is depletion of the C_10_ molecules relative to the C_9_ molecules, which will be discussed further below. In the dimer region, the fraction of the C_19_–C_20_ molecules decreases from 6.8% (without scavenger) to 3.0% (with butanol). The C_20_H_30–34_O_*x*_ fraction measured by the NO_3_-CIMS is depleted (Fig. S2C and D†), which is consistent with previous flow tube studies^18^ and the particle phase composition (Fig. S3†). The C_16_–C_18_ region observes small changes on a relative scale (see Table S1†). Though, considering the mass concentration is lower for the scavenger experiments, the butanol and cyclopentane experiments exhibit lower absolute concentrations of C_16–18_ dimers, while the cyclohexane experiments have no difference relative to the no-scavenger experiment (excluding the mixed dimer products). The depletion of dimers comes from a change in the RO_2_ identities. For instance C_14_ molecules are not observed in the reaction without scavengers, while C_14_ molecules form *via* reactions between butanol radicals and α-pinene radicals. The main RO_2_ radical from the butanol scavenger is C_4_H_9_O_3_.^10,18,26,37,38^ C_4_H_9_O_3_ then reacts with the RO_2_ radicals from α-pinene ozonolysis, C_10_H_15_O_4,6_, to form the dominant C_14_ mixed dimers observed (C_14_H_24_O_5,7_) with an odd number of oxygen atoms. Another possibility to form C_14_ dimers could come from the reaction between the stabilized Criegee intermediate and the scavenger directly (*i.e.* C_10_H_16_O_3_ + C_4_H_10_O),^17,39^ the products of which would form C_14_H_26_O_4_. Based on the concentrations of the butanol and water in the chamber, approximately half of the reactivity of the Criegee should take place with butanol (assuming a reaction rate similar to propanol).^40^ However, C_14_H_26_O_4_ makes up less than 0.01% of the total EESI-TOF signal in the chamber, suggesting this pathway is not significant under our experimental conditions, or the species is too volatile to be in the particle phase. Overall, the formation of C_14_ molecules is a clear indicator that there exist unwanted effects of using scavengers on the chemistry occurring in the chamber.

Fig. 1C and D show that the ‘mixed dimers’ formed from the cycloalkane experiments are C_16_H_26_O_5,7_ and C_15_H_24_O_5,7_ for the cyclohexane and cyclopentane experiments, respectively, and preferentially form with odd-numbered oxygen atoms. If the formation pathway is the same as for butanol, then the molecules with an odd number of oxygen atoms must come from the mixture of an RO_2_ with even number of oxygen atoms + RO_2_ with odd number of oxygen atoms. The dominant α-pinene RO_2_ radicals are C_10_H_15_O_4,6_ and they must combine with either C_6_H_11_O_3_ or C_5_H_9_O_3_, respectively, to form the formula shown above. These formulae differ from the initial scavenger RO_2_ formed from the reaction with an OH radical, which are C_6_H_11_O_2_ (cyclohexane) and C_5_H_9_O_2_ (cyclopentane).^41^ Reaction schemes in the ESI (Schemes S1 and S2†) show how the initial RO_2_ can react with another RO_2_ to form an alkoxy radical, which can rapidly undergo a ring-opening reaction to form a second generation RO_2_ radical. These second-generation RO_2_ radicals (C_6_H_11_O_3_ – cyclohexane and C_5_H_11_O_3_ – cyclopentane) possess a formula matching the expected combination of scavenger and α-pinene oxidation products. An additional aspect in these experiments comes from the formation of dimers that have a carbon number equal to: C_scav_ + C_10_ − 1, which forms C_15_H_24_O_4,6_ in the cyclohexane experiment. In addition, there appears to be a systematic decrease in the C_10_ species with the increasing carbon content of the OH scavenger. For example, the C_10_ fraction decreases from 32% (no scavenger) to 23% (butanol), 12% (cyclopentane), and 9% (cyclohexane), which cannot be explained by changes in mass concentrations. Considering the scavenging of OH is effectively the same in all experiments (with scavengers) and the mass concentrations are similar, the observed differences should be attributed to radical reactions between the oxidation products of the scavengers and α-pinene. One possibility is that the reactions with RO_2_ radicals from cycloalkanes promote reactions *via* the alkoxy pathway which undergo subsequent fragmentation reactions. The cycloalkane experiments also exhibit the formation and inclusion of a small fraction of scavenger dimers (C_12_ – cyclohexane: see Fig. 1D, and C_10_ – cyclopentane: see Fig. S1B†), and small amounts of scavenger oxidation products (Fig. 1C and D), demonstrating three pathways for scavenger inclusions into SOA.

**Fig. 3:**
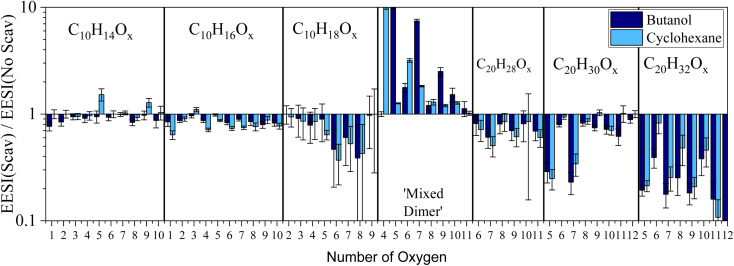
Ratio of gas-phase EESI-TOF signals with scavenger to gas-phase EESI-TOF signal without scavenger observed from the flow tube for specific molecular classes. Mixed dimer contribution is shown as absolute intensity scaled so the maximum intensity is 10 from each experiment. The mixed dimer class corresponds to the C_14_H_24_O_*x*_ (butanol) and C_16_H_26_O_*x*_ (cyclohexane).

We further designed a flow-tube experiment to investigate the formation of the ‘mixed dimers’ in the gas phase from the systems discussed so far. The bar plot of Fig. 3 shows the gas-phase EESI-TOF signal (with scavenger)/EESI-TOF signal (without scavenger) for experiments with butanol and cyclohexane, with a CPC verifying particle number concentration <1 cm^−3^. The ESI (Fig. S4†) includes a time series to demonstrate how the injection of a scavenger influences the gaseous oxidation products in real-time. As we can see from Fig. 3, depletion of C_10_ molecules formed *via* OH chemistry are observed, with C_10_H_16_O_*x*_ depleted by 10–20%, and C_10_H_18_O_*x*_ depleted by up to 60% which is less than the depletion (80–90%) observed in the chamber experiment (Fig. 2). This could result from incomplete mixing in the flow tube, and does not result from changes in gas-particle partitioning due to the lower mass loadings with scavengers (presented in the ESI†). Unfortunately, the small signal-to-noise ratio for the C_10_H_18_O_*x*_ molecules results in relatively large error bars. Depletion of C_20_ dimers occurs with the addition of a scavenger, with C_20_H_32_O_*x*_ being depleted by 90% and exhibiting an even–odd oxygen atom behavior. Dimers with odd number of oxygen atoms are depleted because these molecules are formed from RO_2_–RO_2_ reactions of the OH (C_10_H_17_O_3,5_) and O_3_ reaction pathways (C_10_H_15_O_4,6_). C_20_H_30_O_5,7_ molecules are also depleted by up to 80%, while the rest of the C_20_H_30_O_*x*_ are only slightly diminished (by 30–50%), consistent with.^18^

For the experiments with a butanol scavenger, the main ‘mixed dimers’ formed have an odd number of oxygen atoms (C_14_H_24_O_5,7,9,11_), in good agreement with results from the smog chamber experiments, discussed above. In contrast, there is a difference between flow tube and the smog chamber results for the cyclohexane experiments where the principal number of oxygen atoms for the C_16_H_26_O_*x*_ ‘mixed dimers’ in Fig. 3 are even C_16_H_26_O_4,6,8_, which differs from the results from the smog chamber (odd) in Fig. 1D (C_16_H_26_O_5_ and O_7_). When modelling the oxidation processes with the 0-D box model (F0AM) from the flow tube and the smog chamber, the ratio of the C_6_H_11_O_2_ : C_6_H_11_O_3_ varies substantially between the two experiments (∼20 for smog chamber and 100–300 in the flow tube). The difference in the ratio comes from the time scale of the two experiments, and is not impacted by the concentration difference in the experiments. The initial RO_2_ radicals are still being formed in the flow tube, while longer times in the smog chamber allows the RO_2_ radicals to undergo further reactions (with other RO_2_ or HO_2_ radicals) forming second- and third-generation radicals. Therefore, differences in the ‘mixed dimer’ formed in the flow tube (O_4_ and O_6_) *vs.* the smog chamber (O_5_ and O_7_) reflect the distribution of the scavenger RO_2_ radicals present. Because the main ‘mixed dimer’ formed in the chamber comes from the second-generation scavenger RO_2_ radical (C_6_H_11_O_3_) despite the initial scavenger RO_2_ (C_6_H_11_O_2_) having a larger concentration demonstrates mixed dimer formation is faster between α-pinene-RO_2_ radicals and C_6_H_11_O_3_ when compared to C_6_H_11_O_2_. This is in agreement with the fact that dimer formation rates increase with the increase of the RO_2_ oxygen content.^42,43^

In addition, the extent of C_10_ depletion differs depending on the identity of the scavenger in both the smog chamber (Fig. 1) and flow tube (Fig. 3). When considering the RO_2_ reaction pathways for each scavenger (shown in the ESI†), the fates of the RO_2_ radicals down the alkoxy pathway lead to different results. The butanol derived alkoxy radical terminates with the formation of HO_2_ and acetaldehyde. Scheme S1† shows the cyclohexane RO_2_ radicals going through an alkoxy radical until terminating with an HO_2_ radical. A consequence of the alkoxy pathway can be an enhancement of unimolecular fragmentation products,^19^ resulting in a shift in the carbon distribution away from C_10_ molecules to smaller carbon containing species (C_7–9_). Consequently, there is a shift in the carbon distribution toward smaller carbon containing molecules for all scavengers used in the chamber (Fig. 1) with the most substantial depletion occurring for the cycloalkanes in particular, which supports this explanation.

### 3.2 Modelling and discussion

Some of the changes observed from the use of scavengers comes from changes in the radical balance that occurs. Fig. S4† highlights the reactivity of α-pinene-RO_2_ with HO_2_, and RO_2_ radicals from either the scavenger or α-pinene, assuming general rates of RO_2_ + RO_2_ and RO_2_ + HO_2_ currently used in MCM 3.3.1. These results highlight the importance of HO_2_ in the butanol experiment because of the pathway to form butanal + HO_2_, which promotes RO_2_ radical termination to ROOH monomers over dimer formation. The dimer fraction in Table S1† (and Fig. 1) is roughly similar for all experiments, though the difference in mass loading between experiments will result in a change in the absolute concentration of the dimers. If the EESI results are presented in terms of the total mass flux of the EESI (attograms per second obtained by # s^−1^ × MW × 1 × 10^18^/Avogadro's number) the total dimer signal (C_14–20_) for the no scavenger experiment (4 ag s^−1^) is greater than the absolute dimer signal (2.7 ag s^−1^), consistent with the greater importance of RO_2_ + HO_2_. For the cycloalkane experiments, the absolute concentration of the dimer range (C_14–20_) is not dramatically different to the no-scavenger experiment (cyclopentane – 3.5 ag s^−1^, cyclohexane – 4.2 ag s^−1^), consistent with the importance of RO_2_ + RO_2_ dimer formation on SOA formation. Though, a systematic study on the rates of the RO_2_ + RO_2_ reactions and their branching ratios using a flow reactor would be needed to validate any quantitative modelling of these systems.

Our results raise questions about previous studies that have used OH scavengers to examine SOA physical properties or chemical composition. It also raises the question: why have these products not been previously observed in SOA? Previous measurements of SOA formed in the presence of a scavenger have generally used techniques with harsh ionization processes with substantial fragmentation,^17,34^ or investigations of these molecules have not been a priority when employing offline techniques. Filter sampling techniques can also introduce artefacts and time that affords reactive species to degrade prior to analysis, as has been shown for reactive oxygen species from filter analysis.^44^ Therefore, filter extracts that have measured the chemical composition of SOA may not be an effective method for measuring potentially reactive species formed *via* RO_2_–RO_2_ reactions, including species that hydrolyze in the presence of water or other solvents.

Further, these results show the use of scavengers that form RO_2_ radicals is problematic because it can result in the incorporation of unwanted species into SOA (*e.g.* mixed dimers and scavenger oxidation products), as well as creating a radical environment that dramatically changes the monomer composition of SOA. Without accounting for sensitivity differences of different molecules measured by the EESI-TOF and using purely the relative intensities, shown in Fig. 1, the scavenger incorporated into SOA reaches nearly 20% for cyclohexane, while for the other scavengers the total fraction of artefacts decreases with decreasing carbon number of the scavenger (shown in Table S1†), down to ∼7% for butanol. This accounting includes the increase in C_6_ molecules for the cyclohexane experiment (see Fig. 1A*vs.* 1D) originating from inclusions of cyclohexane oxidation products. Similar increases are also observed in C_12_ molecules and the ‘mixed dimers’ (Fig. 1D). The change in the radical pathways and the incorporation of unwanted scavenger oxidation products in α-pinene SOA demonstrates the necessity to consider which OH scavenger to use and if to use an OH scavenger at all. Additionally, the atmosphere is rife with potential scavengers of OH radicals, and ultimately scavengers used within chambers should effectively mimic atmospheric conditions. Currently, many chamber experiments possess large concentrations of RO_2_ radicals, while in the atmosphere HO_2_ is a significant sink of RO_2_ radicals. Therefore, the goal should be to use scavengers that do not incorporate the scavenger and that produce an atmospherically relevant radical balance (*e.g.* HO_2_*vs.* RO_2_).

**Fig. 4:**
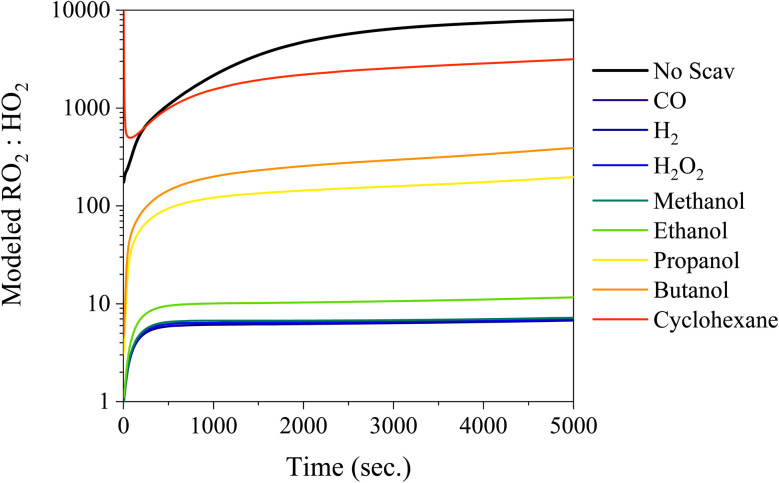
RO_2_ : HO_2_ ratio modelled using a box model based on MCM v3.3.1 for: α-pinene 25 ppb, O_3_ 250 ppb, and excess concentration of the scavenger (CO – 30 000 ppm, H_2_ – 2%, H_2_O_2_ – 200 ppm, methanol – 200 ppm, ethanol – 200 ppm, *n*-propanol – 200 ppm, *n*-butanol – 200 ppm, and cyclohexane – 200 ppm).

Scavengers such as H_2_, CO, and H_2_O_2_ are good candidates because they will only produce HO_2_ radicals, but the drawbacks of these scavengers include the large concentrations required (H_2_ – 2%), potential safety hazards (H_2_ and CO), and the uptake into the particle phase at elevated RH (H_2_O_2_). To probe the impact of the HO_2_ : RO_2_ ratio from different scavengers, a simple chamber box model using MCM^37,38^ simulated the RO_2_ : HO_2_ ratio in the chamber in Fig. 4 (the absolute concentrations are shown in Fig. S5†). For comparison, Fig. 4 also includes other scavengers such as, CO, H_2_, H_2_O_2_, and a series of alcohols, to probe the differences between RO_2_ and HO_2_ concentrations. RO_2_ concentrations are always at least one order of magnitude greater than the HO_2_ concentrations for all conditions explored here. Despite the large concentrations of RO_2_ radicals, the reaction rate between HO_2_–RO_2_ is ∼2 orders of magnitude faster than RO_2_–RO_2_ reactions, consequently HO_2_–RO_2_ will be the dominant reaction pathway when the RO_2_ : HO_2_ is below 100 (*e.g.* CO, H_2_O_2_, H_2_, methanol, and ethanol). Reactions between HO_2_ and RO_2_ will also promote the formation of peroxide functional groups and inhibit formation of dimers *via* the RO_2_–RO_2_ pathway. Additionally, higher concentrations of HO_2_ more realistically capture the HO_2_ : RO_2_ ratio present in the atmosphere as opposed to the RO_2_ dominant chemistry regime typically found in chambers.

In summary, the roles of the scavengers in these experiments are multi-faceted because they influence the HO_2_ : RO_2_ ratio, the identity of the RO_2_ radicals present, and the fate of the RO_2_ radicals. The differences in the types of radicals produced in the gas phase (OH *vs.* HO_2_*vs.* scavenger-RO_2_*vs.* α-pinene-RO_2_) ultimately determines a substantial fraction of the composition of the SOA formed. Given many fundamental studies about SOA are performed in chambers or flow tubes with the presence of a scavenger, it is important to understand the role they will play in the chemistry taking place. This study shows significant changes in composition of α-pinene SOA as a function of OH scavenger and necessitates their further study and consideration.

## Data availability

Data can be found at the Eurochamp Database of Atmospheric Simulation Chamber Studies (https://data.eurochamp.org/).

## Author contributions

Chamber investigations were performed by DB, VP, FL, AB, MX, XZ, and WH. Flow tube studies were investigated by DB, DSW, CPL. JS, AP, IEH and UB obtained funding for this work. DB prepared the manuscript with contributions from all co-authors.

## Conflicts of interest

The authors declare no conflict of interests with the performed work.

## Supplementary Material

EA-003-D2EA00105E-s001
