# Supplementary material for: Effect of OH scavengers on the chemical composition of α-pinene secondary organic aerosol
Source: Environ Sci Atmos. 2022 Nov 4;3(1):115–23. doi: 10.1039/d2ea00105e (PMC9850668; doi:10.1039/d2ea00105e)
Supplement: EA-003-D2EA00105E-s001 [file EA-003-D2EA00105E-s001.pdf]

Electronic Supplementary Material (ESI) for Environmental Science: Atmospheres.
This journal is © The Royal Society of Chemistry 2022

Table S1: Normalized Composition of SOA without and with different scavengers.

	No Scav	Butanol	Cyclopentane	Cyclohexane
Monomer[a]	0.784	0.767	0.635	0.528
C20 - C19	0.068	0.030	0.036	0.036
C18 - C16[b]	0.095	0.091	0.086	0.138
Mixed Dimer	--	0.047	0.079	0.101
Total Scavenger Inclusions[c]	--	0.069	0.166	0.195

[a]Monomer region is designated $C_5 – C_{10}$. **[b]** C_{16} is not included for the cyclohexane experiment. [c] Total scavenger inclusions artefacts are a summation of scavenger dimers (e.g. C_{12} for cyclohexane, and $C_{10}H_{18}O_{5\text{-}10}$ for cyclopentane), and oxidation products of the scavenger (C_6 products of cyclohexane and C_5 products for cyclopentane).

In the smog chamber experiments with OH scavengers the species that were most affected by the scavenger addition were the more highly oxygenated molecules (i.e. #O = 5-8), while the C10H18O4 was effectively unchanged.

To explore if volatility and organic mass concentration drives this effect, Table S2 shows the fraction in the particle phase (F_p) calculated based on the parameterizations in Stolzenburg et al. (2018) to estimate the saturation vapor concentration of each molecule, assuming equilibrium partitioning. The #O = 5 concentrations should be 40% lower in the scavenger experiments, the #O = 6 would be 14% lower, and #O = 7+ would be unaffected. This would not explain the complete removal of the #O = 6-8 in the chamber experiment relative to the flow tube experiment.

Table S2: Expected particle phase fraction

#C	10	10	10	10
#H	18	18	18	18
#O	5	6	7	8
C*	133.35	5.89	0.62	0.04
No Scavenger Avg. Fp (C_{OA} = 25 ug m^{-3})	0.16	0.81	0.98	1.00
Scavenger Avg. Fp (C_{OA} = 14.3 ug m^{-3})	0.10	0.70	0.96	1.00

RO_2 Reaction Pathways

RO_2 → ROOH

RO_2 → RO (carbonyl) + OH

RO_2 + HO_2 → ROOH (hydroperoxide) + O_2

RO_2 + HO_2 → RO + OH + O_2 (alkoxy radical pathway)

RO_2 + $R'O_2$ → ROH (alcohol) + R'O (carbonyl) + O_2 – $R1_1$

RO_2 + $R'O_2$ → RO + R'O + O_2 (alkoxy radical pathway) – $R1_2$

RO_2 + $R'O_2$ → ROOR' + O_2 ("Dimer" formation) – $R1_3$

Scheme 1: MCM Reaction Scheme for Cyclohexane + OH

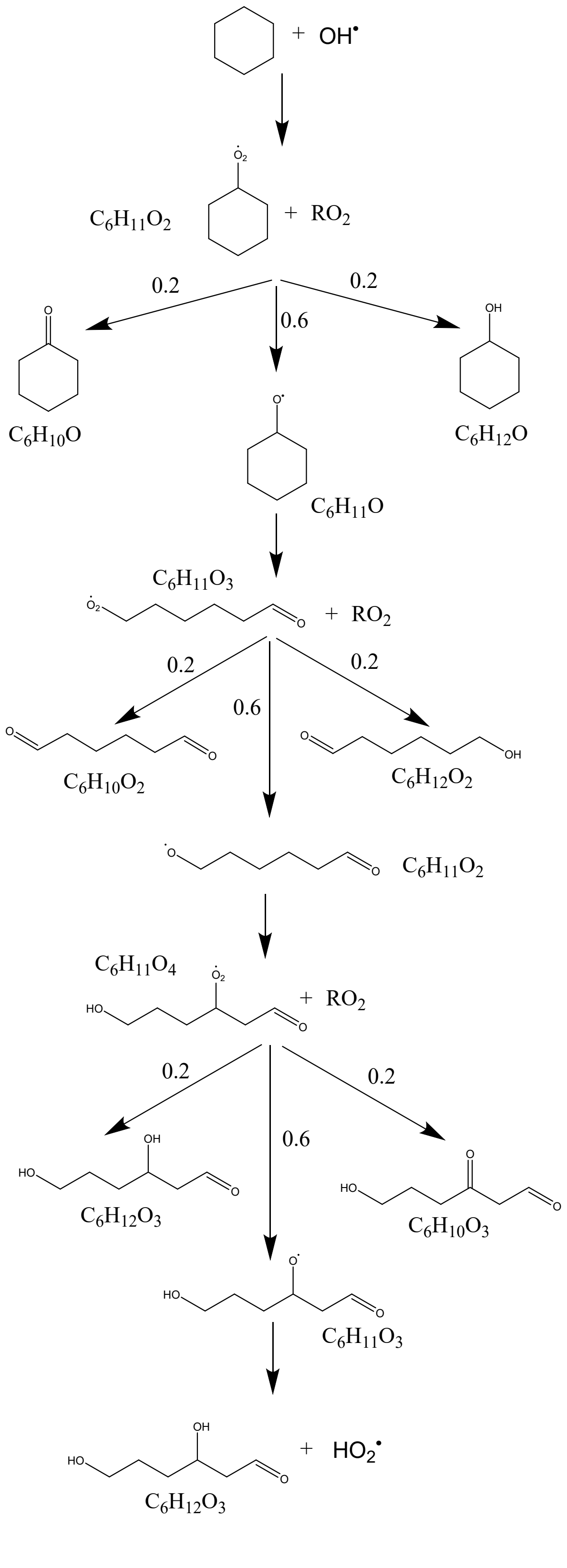

Scheme 2: Reaction Scheme for Butanol + OH

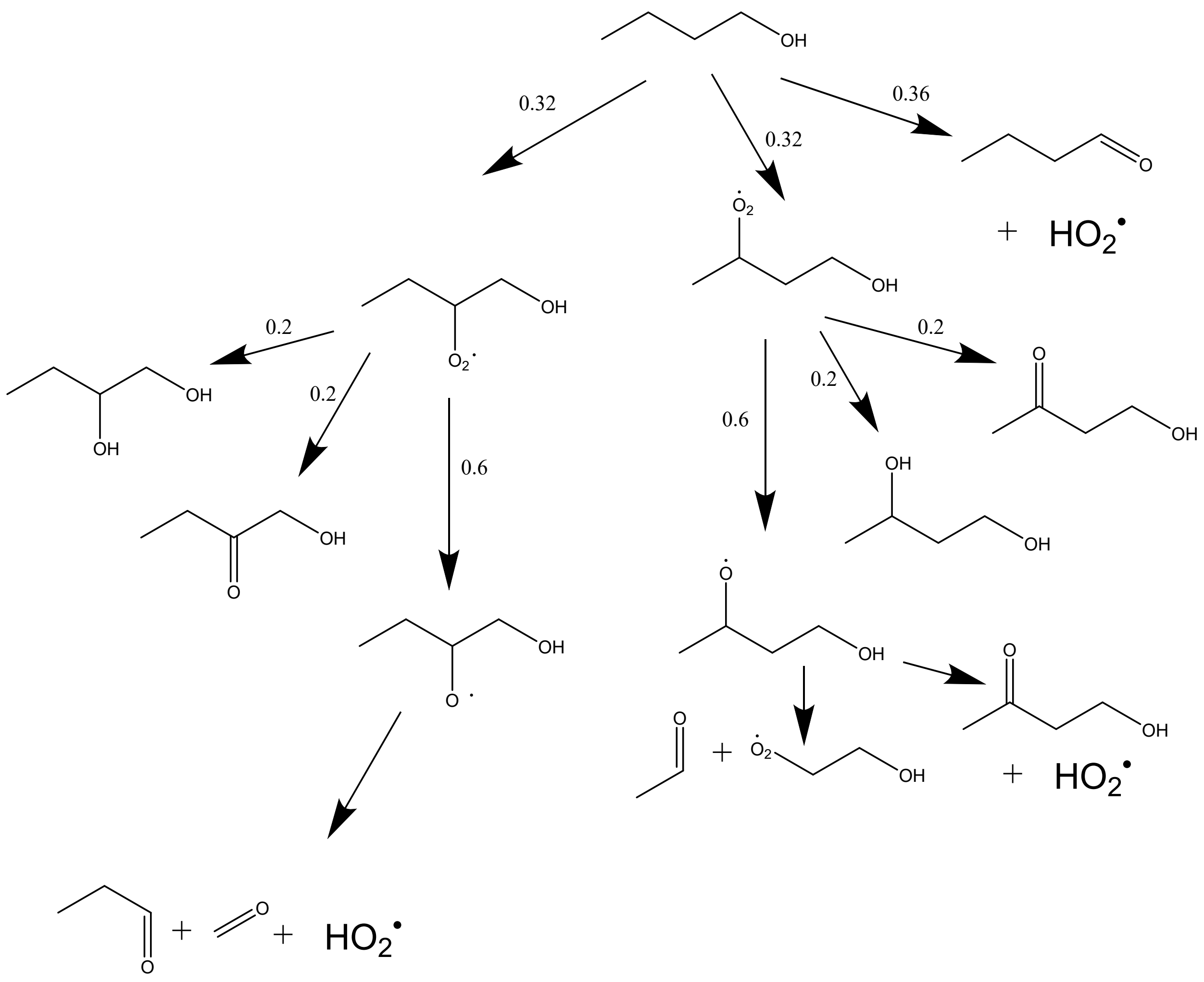

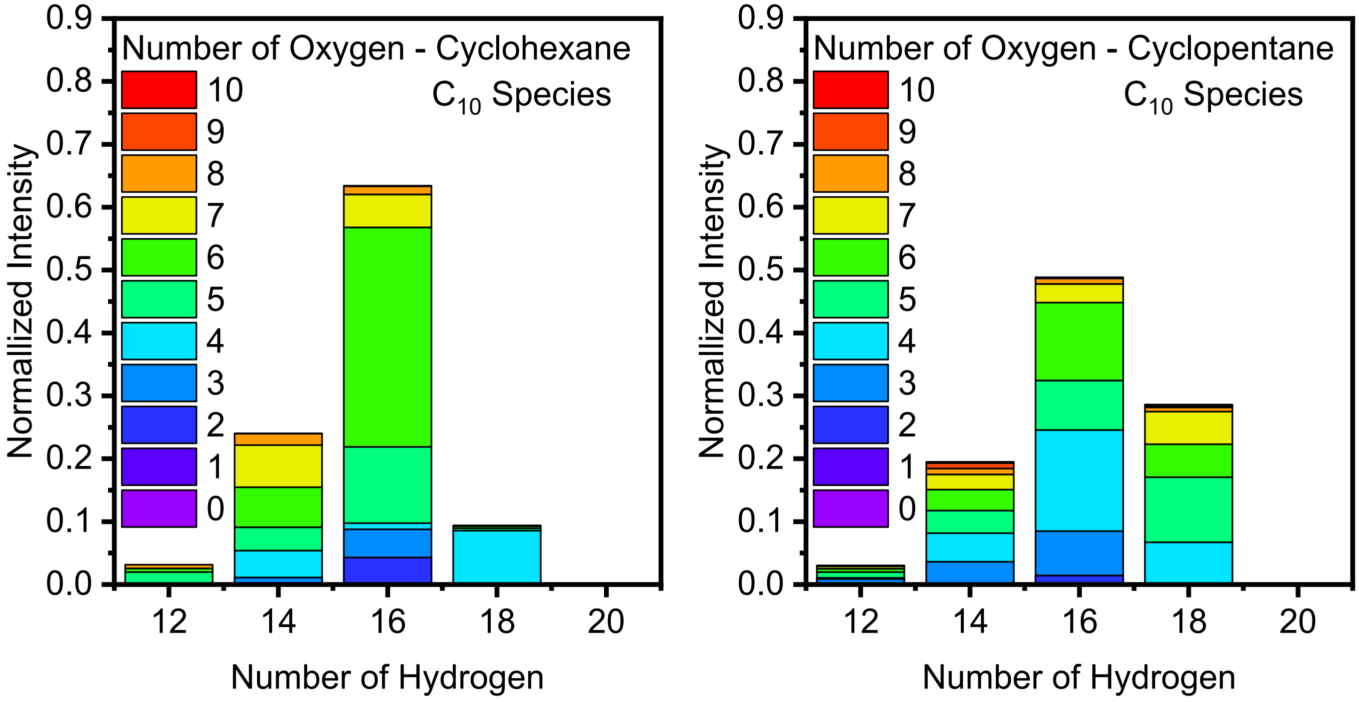

Figure S1A) Hydrogen distribution for C_{10} species stacked according to oxygen number at the beginning of an experiment with cyclohexane as a scavenger. **Figure 1SB)** Hydrogen distribution for C_{10} species stacked according to oxygen number at the beginning of an experiment with cyclopentane as a scavenger. Dimers of cyclopentane oxidation product form $C_{10}H_{18}O_x$ molecules preventing assessment of the α-pinene-derived $C_{10}H_{18}O_x$. For each experiment the distribution is shown 30 min after the injection of α-pinene.

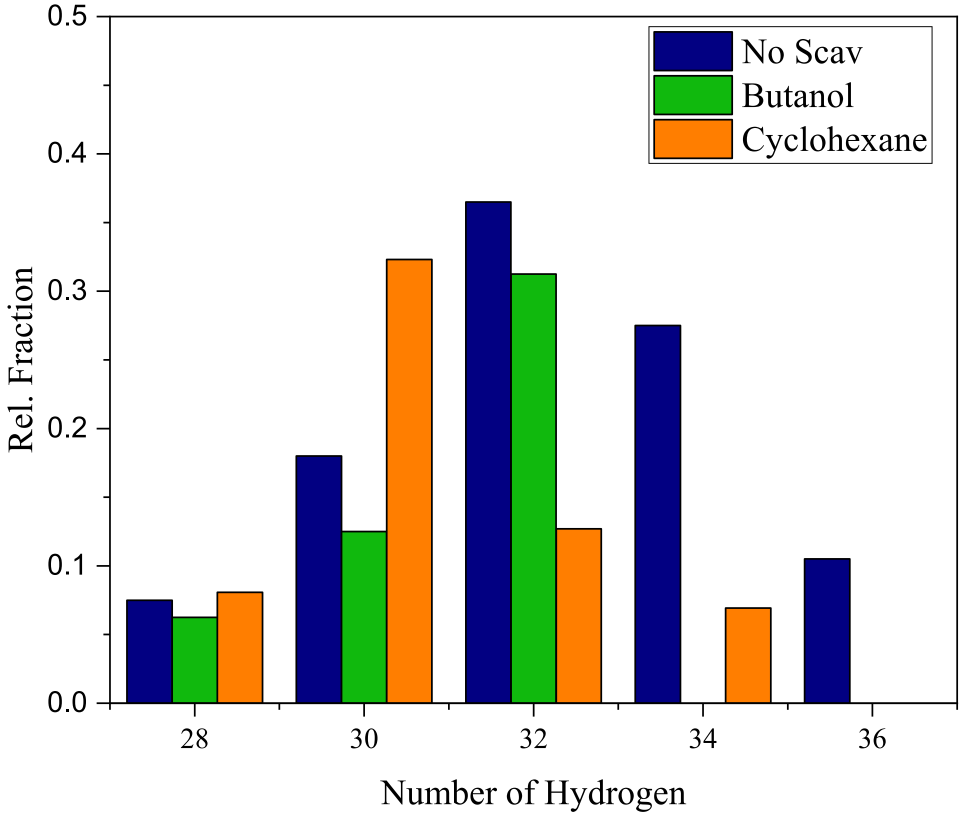

Figure S2: The distribution of the number of hydrogen present in the $C_{20}H_{28\text{-}36}O_x$ molecules present during an experiment with cyclohexane, butanol and no scavenger present; each distribution is obtained at the maximum mass. The $C_{20}H_{32\text{-}36}O_x$ are depleted when an OH scavenger is present because the $C_{10}H_{17}O_x$ RO_2 products are scavenged. In the experiment with scavengers the intensity is scaled according to the depletion in mass relative to the no scavenger experiment.

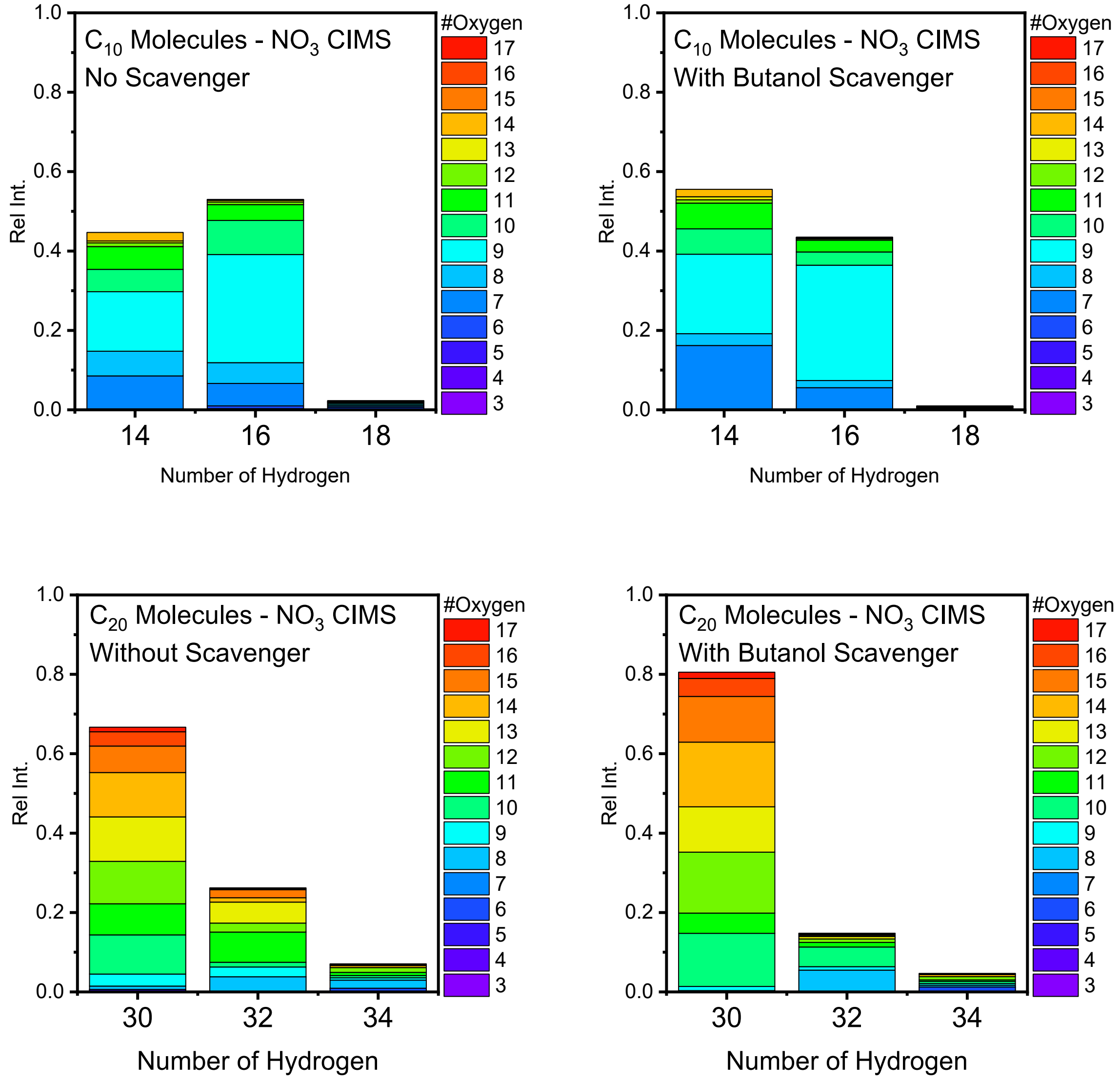

Figure S3A and 3B) NO_3-CIMS data from experiments for C_{10} species without an OH scavenger (**A**) and with butanol as an OH scavenger (**B**) for the shown number of hydrogen and colored by oxygen number. **Figure S3C and 3D)** NO_3-CIMS data from experiments for C_{20} species without an OH scavenger (**C**) and with butanol as an OH scavenger (**D**) for the shown number of hydrogen and colored by oxygen number. All data is averaged over the first 30 min of the experiment because of the low signal in the chamber of these gas phase products. There is a marked decrease in sensitivity for the NO_3-CIMS toward highly oxygenated molecules formed from OH chemistry when compared to O_3 chemistry (Berndt et al., 2016).

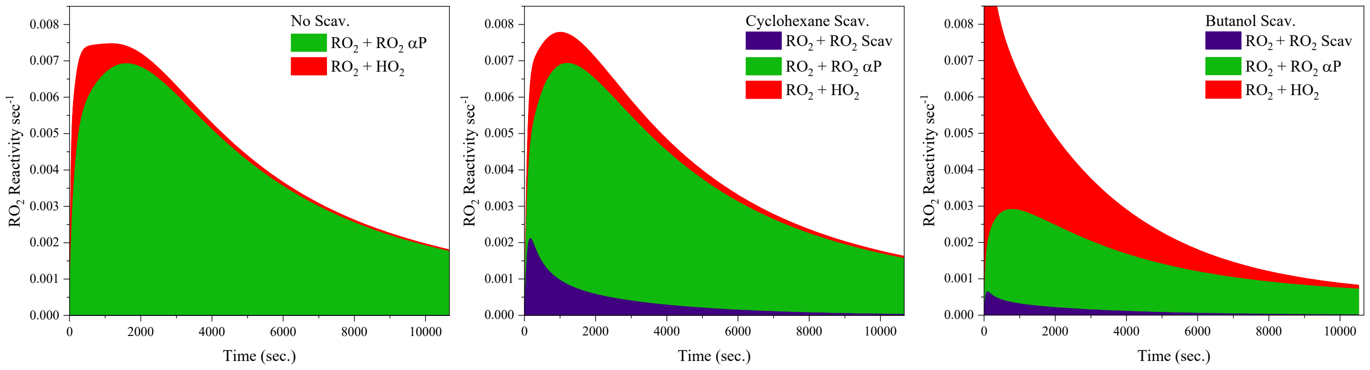

Figure S4: Modelled α –pinene RO_2 reactivity in the experiments with no scavengers, cyclohexane as a scavenger and butanol as a scavenger. The model used 25 ppb α-pinene, 250 ppb O_3, and 200 ppm of scavenger (if needed) as inputs.

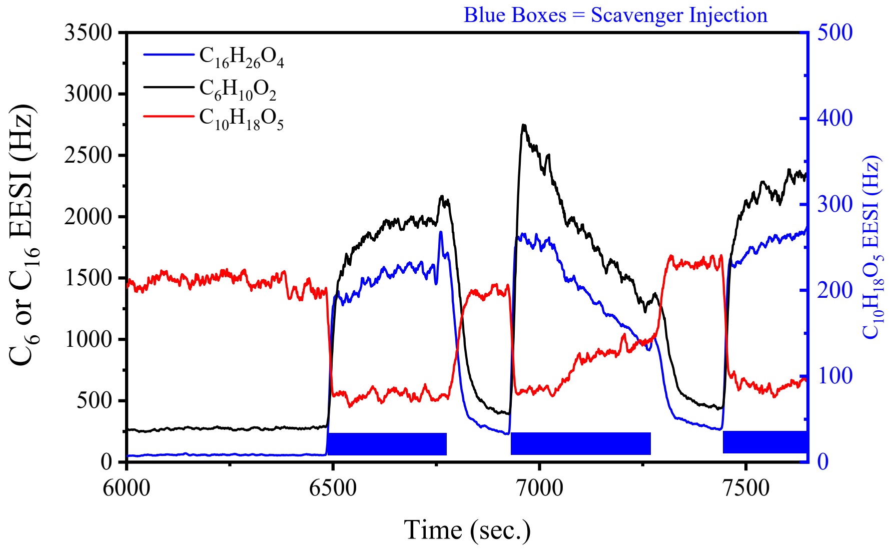

Figure S4) Gas-phase EESI signal for 3 representative molecular formulae for the flow tube experiment with cyclohexane during 3 different additions. The first at *t* = 6500 s the cyclohexane concentration is constant (~200 ppm). At *t* ~6950 s a burst of cyclohexane is added (>500 ppm) and was allowed to steadily decay away. Significant depletion of the OH chemistry product is observed ($C_{10}H_{18}O_5$) coupled with formation of oxidation products from the scavenger ($C_6H_{10}O_2$) and the mixed dimer ($C_{16}H_{26}O_4$).

Comment [BU(]: use same font as above

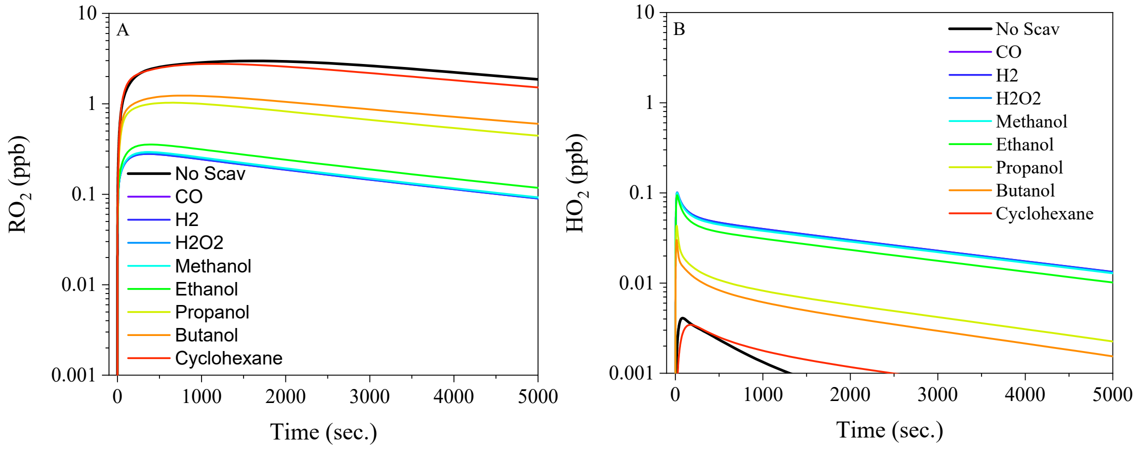

Figure S5) RO_2 **(A)** and HO_2 **(B)** concentrations modelled using a box model based on MCM v3.3.1 for: α-pinene 25 ppb, O_3 250 ppb, and excess concentration of the scavenger (CO – 30,000 ppm, H_2 – 2%, H_2O_2 – 200 ppm, methanol – 200 ppm, ethanol – 200 ppm, n-propanol – 200 ppm, n-butanol – 200 ppm, and cyclohexane – 200 ppm).

References:

Berndt, T., Richters, S., Jokinen, T., Hyttinen, N., Kurtén, T., Otkjær, R. V., Kjaergaard, H. G., Stratmann, F., Herrmann, H., Sipilä, M., Kulmala, M., and Ehn, M.: Hydroxyl radical-induced formation of highly oxidized organic compounds, Nat. Commun., 7, 13677, 10.1038/ncomms13677, 2016.
